# NFAT5 in cellular adaptation to hypertonic stress – regulations and functional significance

**DOI:** 10.1186/1750-2187-8-5

**Published:** 2013-04-23

**Authors:** Chris YK Cheung, Ben CB Ko

**Affiliations:** 1 Department of Anatomical and Cellular Pathology, and The State Key Laboratory in Oncology in South China, The Chinese University of Hong Kong, The Prince of Wales Hospital, Rm 38019, Clinical Sciences Building, Shatin, Hong Kong, China

**Keywords:** NFAT5, Hypertonic stress, Osmoadaptation, OREBP, TonEBP

## Abstract

The Nuclear Factor of Activated T Cells-5 (NFAT5), also known as OREBP or TonEBP, is a member of the nuclear factors of the activated T cells family of transcription factors. It is also the only known tonicity-regulated transcription factor in mammals. NFAT5 was initially known for its role in the hypertonic kidney inner medulla for orchestrating a genetic program to restore the cellular homeostasis. Emerging evidence, however, suggests that NFAT5 might play a more diverse functional role, including a pivotal role in blood pressure regulation and the development of autoimmune diseases. Despite the growing significance of NFAT5 in physiology and diseases, our understanding of how its activity is regulated remains very limited. Furthermore, how changes in tonicities are converted into functional outputs via NFAT5 remains elusive. Therefore, this review aims to summarize our current knowledge on the functional roles of NFAT5 in osmotic stress adaptation and the signaling pathways that regulate its activity.

## The mammalian osmoadaptation response

Exposure of mammalian cells to anisotonic extracellular media results in water flux across the cell membrane due to alternations in extracellular osmolality, leading to an alternation in cell volume. Cells respond to volume changes by the activation of membrane electrolyte transporters which serve to alter the concentration of intracellular solutes and to prevent excessive volume perturbation [1]. For cells subjected to a hypertonic challenge, there is a net efflux of water accompanied by cell shrinkage. Within seconds, charged ions including Na^+^, K^+^ and Cl^-^ are transported into the cells by various electrolyte transporters such as Na^+^-K^+^-Cl cotransporter, Na^+^/H^+^ exchanger and Cl^-^/HCO3^-^ exchanger [2], aiming to equalize the difference in ionic concentration between extracellular and intracellular compartments, and to restore cell volume. The consequence is an increase in intracellular ionic strength and the crowding of intracellular molecules, with various deleterious effects on cell functions. Such effects include the denaturing of macromolecules such as proteins and DNAs [3], the disruption of the mitochondrial structure and functions [4,5], an inhibition of protein translation [6], and an alternation of cytoskeletal architecture [7]. Extracellular tonicity beyond the tolerable limit of cells may also led to apoptotic cell death [8].

**Figure 1:**
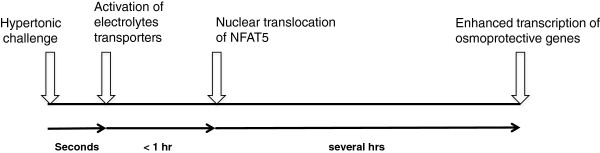
Time frame of short-term and long-term adaptation to hypertonic stress.

To restore biochemical homeostasis under hypertonic stress, cells elicit a genetic program of osmoadaptive responses in which intracellular electrolytes are gradually replaced by uncharged small organic osmolytes including sorbitol, betaine, myo-inositol, taurine and glycerophosphocholine [9,10]. These organic osmolytes play a key role in osmoadaptation because they can be accumulated to a high level without perturbing macromolecular structure and function. Specific enzymes and transporters are responsible for the accumulation of these organic osmolytes: sorbitol and glycerophosphocholine are synthesized by aldose reductase (AR) and neuropathy target esterase (NTE), respectively; whereas betaine, myo-inositol and taurine are taken up into cells via betaine transporter (BGT1), sodium/myo-inositol cotransporter (SMIT), and sodium/chloride-dependent taurine transporter (TAUT), respectively [11,12]. Gene transcription of these enzymes and transporters, collectively known as osmoprotective genes, is markedly upregulated by hypertonic challenge. This is carried out by one or multiple enhancers known as the osmotic-response element (ORE) [13,14] or tonicity-responsive enhancer (TonE) [15] located in the regulatory region of these genes, except for the NTE gene for which the activity of putative OREs has not been functionally confirmed [11]. The identified ORE and TonE share a putative consensus sequence of NGGAAAWDHMC(N) [16]. In general, the ORE/TonE exists singly or in tandem at the proximal promoter region of these genes, but OREs/TonEs can also be scattered along an extended upstream region, as demonstrated by the SMIT gene [17]. The cognate transcription factor for ORE/TonE was independently identified as TonE-binding protein (TonEBP) and ORE-binding protein (OREBP) through yeast one-hybrid screening [18] and affinity chromatography [19], respectively. TonEBP/OREBP exhibits substantial sequence homology to members of the Nuclear Factor of Activated T cells (NFAT) family, and it was therefore independently identified as NFAT5 by using a homology cloning strategy [20]. Figure 1 illustrates the time frame of activation of electrolyte transporters, NFAT5, and the enhanced transcription of osmoprotective genes, respectively. For simplicity we will refer OREBP and TonEBP to NFAT5 in the rest of this review, for it is the gene symbol from the HUGO Gene Nomenclature Committee database.

## Structural arrangement of NFAT5

At present, four human NFAT5 isoforms (isoforms a to d) have been identified [21]. Isoforms a, b, and c are differentiated by the use of an alternative start site at exon 5, 3, and 1, respectively, whereas isoform d encodes the longest protein that includes an additional 18 amino acids from exon 2. In this review the positions of amino acid residues were numbered with reference to the isoform c.

**Figure 2:**
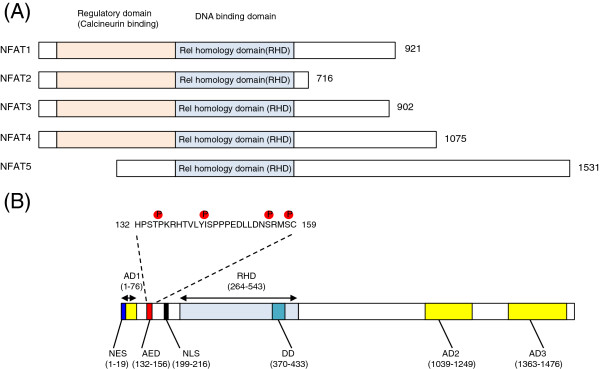
**Members of NFAT family and schematic diagram of NFAT5 functional domains. A**) Alignment of NFAT1-4 with NFAT5. The DNA-binding domains (Rel homology domain) are aligned. The calcineurin-binding domain present in NFAT1-4 is absent in NFAT5. **B**) Overall structure of human NFAT5 protein. Sequence and phosphorylation sites in proximity to the AED are indicated. The corresponding amino acid residues are in parenthesis.

The DNA-binding domain of NFAT5 exhibits sequence homology to the Rel-homology domain (RHD) and it is therefore categorized as the Rel family of transcription factors, which in this family also includes NFAT proteins (NFAT1-4) and NF-kappaB [20,22]. However, the structural similarity between NFAT5 to other NFAT proteins is very low outside the DNA-binding domain. NFAT5 lacks the calcineurin-binding domain that is essential for the activation of NFAT1-4. It also lacks the structural domain found in other NFATs required for the formation of cooperative complexes with Fos and Jun [20,22]. Therefore, NFAT5 is regarded as a distant member of the Rel protein family (Figure 2A). Structurally, in addition to the RHD, NFAT5 contains a canonical nuclear export signal (NES, amino acids 1–19), a consensus bipartite nuclear localization signal (NLS, amino acids 199–216), an auxiliary export domain (AED, amino acids 132–156), a dimerization domain (DD) (amino acids 370–433) within the RHD (amino acids 264–543), and transactivation domain (AD) at the N- (AD1, amino acids 1–76) and C-terminal (AD2, amino acids 1039–1249, and AD3, amino acids 1363–1476), respectively (Figure 2B) [23-25].

## Tonicity-dependent and -independent function of NFAT5

The kidney inner medulla is the only anatomical site where cells are routinely exposed to high and varied levels of sodium and urea due to the operation of urinary concentrating mechanisms. Therefore, the NFAT5-dependent expression of AR [26], BGT1 [27], SMIT [28], and TAUT [29] genes is especially important for the survival of cells at this location. Moreover, NFAT5 is also responsible for the hypertonic induction of heat shock proteins HSP70 [30] and Osp94 [31], where HSP70 was shown to be a molecular chaperone essential for cell survival under hypertonic stress [32], and Osp94 is a member of the HSP110 gene family [33]. Besides osmoadaptive responses, NFAT5 has been implicated in urine concentrating mechanisms in the inner medulla via regulating the expression of the urea transporter (UT-A), aquaporin 1 (AQP1) and aquaporin 2 (AQP2), respectively [34-37]. In addition, NFAT5 is implicated in dehydration natriuresis via regulating the expression of serum- and glucocorticoid-inducible kinase (Sgk1) [38]. NFAT5-deficient mice develop renal atrophy and most of the mice died either after E14.5 or at around 10 days after birth [26].

Interestingly, NFAT5 is also expressed in many non-renal tissues or cells that presumably are well-protected by isotonic body fluids under physiological conditions in which osmoadaptation is deemed unnecessary [39]. However, emerging evidence suggests that certain tissue micro-environments may be exposed to a various extent to hypertonic stress [40,41]. This may explain why NFAT5-dependent osmoadaptive responses are conserved in different cell lines of non-renal origin. In addition, NFAT5 may direct cell-specific gene transcription programs in response to hypertonic stress. For example, in liver cells, NFAT5 induces the expression of CYP2E1 and CYP3A7 of the cytochrome P450 family [42,43]; whereas in T cells, NFAT5 regulates the expression of amino acid transporter ATA2 [44], cell adhesion molecule CD24 [45], TNF-α and lymphotoxin-β, respectively [25,46]. In addition, hypertonicity also regulates the NFAT5-dependent expression of cytokine B cell-activating factor (BAFF) in B cells [47], β1,3-Glucuronosyltransferase-I expression in nucleus pulposus cells [48], monocyte chemo-attractant protein-1 (MCP-1) expression in mesothelial and renal proximal tubular cells [49,50], and VEGFC expression in macrophages [40], respectively. Most recently, NFAT5 has been implicated in the production of T_H_17 cells, a putative target in the development of autoimmune diseases, via the regulation of Sgk1 expression [51]. On the other hand, NFAT5 can be activated in a tonicity-independent manner during T cells activation [39] and skeletal muscle myogenesis [52]. Besides, in macrophages, NFAT5 regulates HIV replication [53], as well as iNOS and IL6 expressions upon activation of a toll-like receptor [54]. Furthermore, cardiac development and function were impaired in NFAT5 knockout mice [55], and NFAT5^+/−^ mice are also are more susceptible to ischemic brain injuries [56]. These findings suggest that besides osmoadaptation, NFAT5 might play a broader role in development, immune function, and cellular stress responses. The tonicity-dependent NFAT5-regulated genes are summarized in Table 1.

**Table 1:** Molecular targets of NFAT5

**Types**	**Name**	**Function**	Evidences (R/E/C/I)*	**Refs.**
**Transporters**	Betaine/γ-aminobutyric acid transporter (BGT1)	Uptake of betaine.	R, E	[15,18,27]
	Sodium dependent myo-inositol transporter (SMIT)	Uptake of myo-inositiol.	R, E, I	[17,28]
	Taurine transporter (TAUT)	Uptake of taurine.	R, I	[29]
	Urea transporter (UT-A)	Transport urea.	R, E, I	[34,36]
	Aquaporin 1 (AQP1)	Water channel.	R, C, I	[35]
	Aquaporin 2 (AQP2)		R, C, I	[36,37,57]
	Aquaporin 4 (AQP4)		C, I	[58]
**Kinases**	Sgk1	Involved in dehydration natriuresis.	R, E, C, I	[38,51]
**Chaperones**	HSP70	Heat shock protein	R, E, I	[28,30]
**Enzymes**	Aldose reductase (AR)	Converts glucose to sorbitol.	R, I	[19,26,28]
	β1,3-Glucuronosyltransferase-I	Synthesis of Glycosaminoglycan	R, C, I	[48]
	Aromatic l-amino acid decarboxylase (AAD)	Synthesis of dopamine.	R, E, I	[59]
	Inducible nitric oxide synthase (iNOS)	Synthesis of nitric oxide.	R, C, I	[54]
**Cytochrome**	CYP2E1	Metabolism of xenobiotics.	R, E, I	[42]
	CYP3A7		R, E, I	[43]
**Cell adhesion molecules**	CD24	T cell proliferation and conversion to memory cells.	C, I	[45]
**Cytokines**	TNF-α	Cytokines.	E, C, I	[25,46]
	IL-6		R, C, I	[54]
	Monocyte chemoattractant protein-1 (MCP-1)	Recruitment of monocytes and T cells to inflammatory region.	R, E, I	[49,50]
**Growth factors**	VEGFC	Lymphangiogenesis	E, C	[40]
**Others**	S100A4/metastasin-1	Cell motility and tumor metastasis.	R, C, I	[60-62]

*R – Reporter gene assay, E – EMSA, C – ChIP, I – Inhibition of NFAT5 (gene knockdown/gene knockout).

## Regulation of NFAT5 activity – mechanisms and signaling pathways

NFAT5 activity is regulated by a tonicity-dependent and -independent manner. In this review we will focus on tonicity-dependent regulation. For tonicity-independent regulation of NFAT5 readers can refer to a recent review by Halterman *et al.*[63].

NFAT5 is regulated in multiple levels in response to changes in extracellular tonicity. These include the control of nuclear abundance, the regulation of transcriptional activity, and the regulation of synthesis. Evidence suggests that the coordinated action of these three mechanisms is important for proper osmoadaptive responses.

## Regulation of nuclear abundance

Under isotonic conditions NFAT5 is not in a static state but undergoes active nucleocytoplasmic shuttling [23]. Therefore the transcription factor is found in both the cytoplasm and nucleus [18,25]. Isotonic nucleocytoplasmic shuttling requires the presence of NES and NLS. On the other hand, changes in extracellular tonicities alter the nuclear abundance of NFAT5 via nucleocytoplasmic trafficking mechanisms. This regulation is rapid [23,64] and it is carried out in a bi-directional manner, in which hypertonic stress increases nuclear import and the accumulation of the transcription factor, whereas hypotonicity leads to its nuclear export [19,64]. Different from isotonic nucleocytoplasmic shuttling, the hypotonic export of NFAT5 requires the presence of AED but not of NES.

The NES located at the first 19 amino acids of the transcription factor is responsible for the nuclear export. It is characterized by a stretch of leucine and isoleucine residues that share significant homology to other canonical NES [23]. Accordingly, leucine-to-alanine substitutions of the NES, or the pharmacological inhibition of the NES export receptor exportin-1 [65], leads to nuclear accumulation of NFAT5 under isotonic conditions [23,66]. On the other hand, the nuclear import of NFAT5 is mediated by the NLS. Although the NLS of NFAT5 contains two clusters of basic amino acids which share sequence homology to the consensus bipartite NLS, only alanine substitution of the first cluster of basic amino acids (amino acids 202–204) blocked the nuclear import of NFAT5 [23]. Therefore, the NLS of NFAT5 belongs to the monopartite NLS similar to that found in the SV40 T antigen. Mutation of the NLS at the first basic amino acids cluster completely blocks the NFAT5 nuclear import both under isotonic and hypertonic conditions [23].

Whereas the NES is required for the NFAT5 nuclear export during isotonic nucleocytoplasmic trafficking, it is dispensable for the nuclear export under hypotonic conditions. Depletion mapping reveals that a second protein domain, named AED, regulates NFAT5 nuclear export under hypotonic conditions. However, AED does not show any sequence homology to other proteins, and was unable to confer nuclear export activity to a fused heterologous protein [23], thus suggesting that the AED may act as a regulatory domain instead of being recognized directly by an export receptor. This notion was supported by the findings that a number of amino acid residues within and in close proximity to the AED were subjected to phosphorylations, which in turns control nucleocytoplasmic trafficking upon changes in tonicity [67]. For example, the sequential phosphorylation of Ser155 and Ser158 by a yet-unknown kinase and casein kinase I respectively is implicated in the hypotonicity-induced nuclear export of NFAT5 [68], whereas the phosphorylations of Thr135 and Tyr143 by CDK5 [67] and c-Abl [69] respectively has been implicated in nuclear import. The mechanism of phosphorylation-dependent nuclear import remains unknown, but recent evidence suggests that phospholipase C-γ1 (PLC-γ1) may play a role by interacting with phosphorylated Tyr143 [70]. In conclusion, it is likely that differential phosphorylation of different amino acid residues works in concert to regulate the nucleocytoplasmic trafficking process. Nevertheless, the way in which different phosphorylations are coordinated and the functional role of each of the phosphorylated residue remains to be characterized.

In a different manner to established notions, the results from the Kwon laboratory [71] suggested that a minimal NLS is solely responsible for NFAT5 nucleocytoplasmic trafficking. Their study showed that the “minimal” NLS composed of 17 amino acids (amino acids 199–215) is responsible both for the nuclear import and export of NFAT5, and this sequence alone is sufficient to direct tonicity-dependent nucleocytoplasmic trafficking of a heterologous protein. Nevertheless, this set of data should be interpreted with caution. It is well conceived that under hypertonic stress there will be distinctive and dominant nuclear localization of NFAT5 (either endogenous or ectopically expressed) in the majority (> 80%) of the cell population [23,25]. This observation is also confirmed by the Kwon laboratory in the same paper in which they described the “minimal” NLS [71]. However, with the minimal NLS-GFP (3GFP-NLS) fusion protein which they used to demonstrate the nucleocytoplasmic trafficking property of the “minimal” NLS, only ~30% of the cell population showed exclusive nuclear localization of GFP signals under hypertonic conditions, and a barely ~10% increase when compared to the cells exposed to isotonicity [71]. Therefore, this “minimal” NLS sequence was not even capable of conferring dominant nuclear import property to a heterologous protein, let alone of acting as a nuclear export signal.

A recent study has revealed an alternative mechanism for the regulation of NFAT5 nucleocytoplasmic trafficking. This study showed that the shortest isoform of NFAT5 (isoform a, which does not contain NES) is anchored to the plasma membrane via myristoylation and palmitoylation at the second (Gly2) and fifth (Cys5) residue of the protein respectively under a resting state, but that it undergoes nuclear import upon hypertonic induction [72]. How this NFAT5 isoform is detached from the plasma membrane upon hypertonic stress remains unknown, but it might represent an alternative mechanism to limit the nuclear import of this NES-deficient NFAT5 isoform to prevent aberrant gene transcription. Nevertheless, at present we do not have information regarding the function of different NFAT5 isoforms, and neither do we know why differential regulation of these isoforms is necessary.

Apart from active nuclear import, nuclear retention appears to be another regulatory strategy for regulating NFAT5 activity under hypertonic stress. The Nup88, a component of the nuclear pore complex at the nuclear membrane [73], was shown to play a role in the regulation of NFAT5 activity in IMCD3 cells via retaining the transcription factor in the nucleus [66]. However, it may be a cell-type specific mechanism because another study has shown that Nup88 does not play a role in the regulation of NFAT5 activity in HEK293 cells [74]. Alternatively, this study also showed that the mutation of DNA contact sites reduces NFAT5 nuclear localization, thereby suggesting that specific binding of NFAT5 to the target gene may be a mechanism for nuclear retention of the transcription factor. Consistent with this notion, the chromatin that flanks the OREs of the aldose reductase gene contains dynamic nucleosomes that are subjected to rapid and reversible loss under hypertonic conditions [75]. NFAT5 dimer binds to DNA by complete encirclement, in which the size of the lumen is sufficient only to accommodate naked DNA [76]. Loss of nucleosome might therefore ensure tight NFAT5-DNA binding for kinetic stability.

## Regulation of transactivation activity

All three transactivation domains (AD1, AD2 and AD3) contribute to the activity of NFAT5. Although each of them enhances gene transcription activity when fused to a heterologous DNA-binding domain alone, only the activity of AD2 is subjected to regulation by changes in tonicities [24]. The ADs are constitutively phosphorylated under isotonic conditions, but whether there is an association between the phosphorylation level and tonicity remains debatable. A study that determined the band-shift of the activation domain in the presence or absence of phosphatase treatment concluded that hypertonic stress enhances the phosphorylation level of the C-terminal transactivation domain (TAD, amino acids 548–1531, which comprises of AD2 and AD3) [77], whereas another study that measured the *in vivo* incorporation of radioactive ^32^P to the ADs suggested that the phosphorylation level of the ADs, including AD2, does not correlate with extracellular tonicities [24]. The discrepancy may be ascribed to the fact that the transactivation domains are constitutively phosphorylated, but that the assays are not equally sensitive for detecting differential phosphorylation of a small number of residues by changes in tonicities. A number of putative phosphorylation sites on TAD was suggested to play a role in regulating NFAT5 activity [78], but direct evidence that these sites are phosphorylated is lacking, and mutational substitution of these sites leads to only a modest reduction in NFAT5 activity.

Apparently, NFAT5 transactivation activity is regulated by a complex mechanism. A number of signaling molecules, including PKA [79], ATM [78], c-Abl [69], HSP90 [80], PARP-1 [80], and MDC1 [81], respectively, are found to be associated with NFAT5 and implicated in the regulation of its transactivation. Furthermore, reactive oxygen species [82], PI3K [83], Fyn and p38 [84], as well as a Rac1/PLC-γ1 signaling cascade [85], may act as more upstream regulators. Although how these molecules are coordinated and regulated during hypertonic signaling remains elusive, the Fyn- and p38-signaling cascades are likely to play important roles in NFAT5 activation; this is because NFAT5 activation and target gene induction could be almost completely abolished by simultaneous deletion/inhibition of p38 and Fyn. On the other hand, a recent study showed that hypertonicity enhances the association between Transcriptional Co-activator with PDZ-binding Motif (TAZ) and NFAT5, leading to the suppression of its DNA-binding and transcriptional activity [86]. This finding raised an intriguing hypothesis that NFAT5 is subjected to negative regulation under hypertonic conditions, where it was supposed to be fully activated. Although such regulatory mechanism might be important for the fine-tuning of NFAT5 activity, the physiological significance of this phenomenon remains to be explored.

## Regulation of synthesis

While nuclear import of NFAT5 could ensure a prompt response for gene transcriptions, an increase in NFAT5 synthesis is considered important for sustaining the genetic program of osmoadaptation under chronic hypertonic stress. This notion is supported by the finding that an increase in NFAT5 mRNA and protein levels was observed at hours after hypertonic challenge [64]. The underlying mechanisms that regulate increased NFAT5 mRNA and protein abundance are still far from clear. Earlier work showed neither an increase in the mRNA transcription rate nor an enhanced mRNA stability of NFAT5 by hypertonicity [64]. Subsequent studies suggested that the mRNA was stabilized via the 5’-UTR [87]. Nevertheless, more recent data has shown that microRNA-dependent mechanisms are involved in the regulation of NFAT5 mRNA stability and protein synthesis. The miR-200b and miR-717 were found to be downregulated upon hypertonic exposure, leading to the increased stability of NFAT5 mRNA and enhanced protein synthesis [88]. It remains to be determined whether the miRNAs act in general or in a cell-specific fashion to enhance NFAT5 mRNA and protein remains. On the other hand, a number of membrane proteins and signaling pathways, such as integrin α_1_β_1_[89], Brx/JIP4/p38 signaling cascade [47], EGF receptor [90], and Na^+^-K^+^-2Cl^-^ cotransporter type 2 (NKCC2) isoforms [91], are implicated in regulating NFAT5 abundance. It will be interesting to see whether and how these pathways are connected, and to elucidate in detail how these molecules transduce osmotic signals to activate the transcription factor.

## Concluding remarks

Despite the passing of more than a decade since the identification of NFAT5 as a central regulator of cellular osmoadaptive responses, we are still at the dawn of understanding the function and regulation of this intriguing transcription factor. Although an increasing number of molecules are being suggested as playing a role in the regulation of NFAT5 activity, evidence and models regarding how it is regulated are still lacking. Importantly, our current knowledge of nucleocytoplasmic trafficking mechanism has fallen short of explaining how NFAT5 is transported between cytoplasmic and nuclear compartments. Neither do we have evidence regarding how changes in tonicities are detected and transduced to the transcription factor for functional output, nor do we know the transcriptional complex required for NFAT5-dependent gene transcriptions. On the other hand, emerging evidence suggests that NFAT5 may play a broader functional role beyond osmoadaptation in a tissue-specific manner. Therefore, we believe that to continue to elucidate the function and regulation of this transcription factor may reveal novel cellular mechanisms for osmoadaptation, and will also give insights into the novel functional role of this transcription factor.

## Abbreviations

AR: Aldose Reductase; NTE: Neuropathy Target Esterase; BGT1: Betaine Transporter; SMIT: Sodium/Myo-inositol Cotransporter; TAUT: Sodium/Chloride-Dependent Taurine Transporter; ORE: Osmotic-response Element; TonE: Tonicity-responsive Enhancer; NFAT5: Nuclear Factor of Activated T Cells; TonEBP: TonE-Binding Protein; OREBP: ORE-Binding Protein; HSP70: Heat Shock Protein 70; UT-A: Urea Transporter; AQP1: Aquaporin 1; AQP2: Aquaporin 2; Sgk1: Serum- and Glucocorticoid-inducible Kinase; TNF-α: Tumor Necrosis Factor-alpha; MCP-1: Monocyte Chemoattractant Protein-1; VEGFC: Vascular Endothelial Growth Factor-C; RHD: Rel-homology domain; NES: Nuclear Export Signal; NLS: Nuclear Localization Signal; AED: Auxiliary Export Domain; DD: Dimerization Domain; AD: Transactivation Domain; CDK5: Cyclin-dependent Kinase 5; c-Abl: V-abl Abelson Murine Leukemia Viral Oncogene Homolog 1; PLC-γ1: Phospholipase C-γ1; PKA: Protein kinase A; ATM: Ataxia telangiectasia-mutated; MDC1: DNA Damage Checkpoint 1; PARP-1: Poly[ADP-ribose] polymerase 1; NKCC2: Na^+^-K^+^-2Cl^-^ cotransporter type 2; EMSA: Electrophoretic mobility shift assay; ChIP: Chromatin immunoprecipitation.

## Competing interests

The authors declare that they have no competing interests.

## Authors’ contributions

CC contributed to reviewing the literature in this review and helped to prepare the manuscript and figures. BCBK drafted the manuscript and approved it in its final form. Both authors read and approved the final manuscript.
